# Relative brain signature: a population-based feature extraction procedure to identify functional biomarkers in the brain of alcoholics

**DOI:** 10.1002/brb3.335

**Published:** 2015-05-23

**Authors:** Nader Karamzadeh, Yasaman Ardeshirpour, Matthew Kellman, Fatima Chowdhry, Afrouz Anderson, David Chorlian, Edward Wegman, Amir Gandjbakhche

**Affiliations:** 1 School of Physics, Astronomy, and Computational Sciences, George Mason UniversityFairfax, Virginia; 2 National Institute of Child Health and Human Development, National Institutes of HealthBethesda, Maryland; 3 Henri Begleiter Neurodynamics Laboratory, Department of Psychiatry, SUNY Downstate Medical CenterBrooklyn, New York

**Keywords:** Alcoholism, classification, electroencephalography's, functional biomarker

## Abstract

**Background:**

A novel feature extraction technique, Relative-Brain-Signature (RBS), which characterizes subjects' relationship to populations with distinctive neuronal activity, is presented. The proposed method transforms a set of Electroencephalography's (EEG) time series in high dimensional space to a space of fewer dimensions by projecting time series onto orthogonal subspaces.

**Methods:**

We apply our technique to an EEG data set of 77 abstinent alcoholics and 43 control subjects. To characterize subjects' relationship to the alcoholic and control populations, one RBS vector with respect to the alcoholic and one with respect to the control population is constructed. We used the extracted RBS vectors to identify functional biomarkers over the brain of alcoholics. To achieve this goal, the classification algorithm was used to categorize subjects into alcoholics and controls, which resulted in 78% accuracy.

**Results and Conclusions:**

Using the results of the classification, regions with distinctive functionality in alcoholic subjects are detected. These affected regions, with respect to their spatial extent, are frontal, anterior frontal, centro-parietal, parieto-occiptal, and occipital lobes. The distribution of these regions over the scalp indicates that the impact of the alcohol in the cerebral cortex of the alcoholics is spatially diffuse. Our finding suggests that these regions engage more of the right hemisphere relative to the left hemisphere of the alcoholics' brain.

## Introduction

A number of imaging modalities, each offering their own advantages and disadvantages, have been employed to investigate the functional and structural effects of alcohol on the brain (Wit et al. 1990; Laakso et al. 2000; Pfefferbaum et al. 2001a; Enoch et al. 2008). Magnetic Resonance Imaging (MRI) and functional MRI (fMRI) are commonly used to explore structural and functional changes in the brain of alcoholics (Hommer et al. 1996; Pfefferbaum et al. 2001b; Tapert et al. 2001; Desmond et al. 2003), respectively. fMRI measures brain's neural activity through its localized hemodynamic response and provides high spatial resolution. Identifying abnormalities in the brain function of alcoholics has been also addressed by less expensive and more portable neuroimaging modality such as functional near Infrared Spectroscopy (fNIRS) (Villringer and Chance 1997; Amyot et al. 2012; Bunce et al. 2013) which measures local hemodynamic changes over the cerebral cortex (Felleman and Van Essen 1991). On the other hand, the capability of imaging modalities such as Magnetoencephalography (MEG) (Hämäläinen et al. 1993) or electroencephalography (EEG) (Niedermeyer and da Silva 2005) that are capable of measuring neuronal activity directly with high temporal resolutions (and low spatial resolution) has been less explored. Electroencephalography, which captures neuronal activity in the range of milliseconds can be employed to capture neural dysfunction that occur in the short-lasting temporal window (Karamzadeh et al. 2013). In particular, the Event-Related-Potentials (ERP) consisting of a number of elicited peaks (ERP components), manifest regional transient variations after a stimulus is presented. These peaks are known to be related to a wide variety of sensory, cognitive or motor events. Therefore, they are commonly used to study a number of brain dysfunctions (Pfefferbaum et al. 2001b; Balan et al. 2002; Mathalon et al. 2002). To capture brain potential impacts of long-term alcohol consumption, analysis of ERP signals has been bounded to the certain temporal windows of the signals (such as P300 that occurs 300–500 ms after the stimulus onset) (Begleiter and Porjesz 1999) or transforming signals to the frequency domain to explore specific frequency ranges (Rangaswamy et al. 2002). These studies attempt to study the characteristics of ERP's amplitude fluctuations (Bostanov 2004), power spectral density (Musha et al. 1997), or time-frequency analysis (Wang et al. 2004), to extract informative and discriminative features. A major challenge in EEG data analysis is its high dimensionality. The high dimensionality of the EEG data adds more complexity, increases the computational time, and complicates the data analysis due to the phenomenon called “curse of dimensionality” (Lotte et al. 2007). Although the aforementioned feature extraction techniques reduce the dimensionality of the EEG signals, yet the feature space corresponding to a single subject is relatively high due to the large number of signals recorded from the subject's brain. Therefore, the EEG feature extraction is typically followed up by a channel selection procedure (Ansari Asl et al. 2007) that selectively discards signals corresponding to certain channels of the subjects' EEG signals. Furthermore, the common EEG feature extraction techniques are not designed to include attributes regarding the subject's population association into their process of feature extraction and solely extracts information from individual's EEG signals.

In this work, we introduce and validate a novel time series feature extraction technique, Relative Brain Signature (RBS) that can be utilized to provide an effective dimensionality reduction for ERP signals, which does not require the typical channel selection procedure and accounts for all the ERP signals. Furthermore, unlike common feature extraction techniques that do not consider subject's population association in the procedure of feature extraction, RBS technique obtains information from subjects' ERPs by investigating their relationship to the given populations of the study. RBS combines vector space analysis and orthogonal subspace projection to generate the feature vector that describes the relationship between a subject and populations. We show that our proposed technique can be used to identify the functional biomarkers related to a specific population. We also provide a topographic map of the localized biomarkers.

## Methods

### Experimental paradigms and data acquisition

We assessed our method of extracting RBS using an EEG data set collected from two populations of alcoholic and control subjects. These data were collected by the Neurodynamics Laboratory, SUNY Downstate Medical Center, (supported by NIH grants AA05524 and AA026686) from a group of alcoholics and control subjects and was first published in Zhang et al. (1995). The control group consisted of 43 right-handed male (the data set originally contained 45 subjects of which two subject data were excluded to file errors and empty trials) subjects with an age range of 19.4–38.6 years. The alcoholic group consisted of 77 males with an age range of 22.3–49.8 years. Alcoholic subjects were initially diagnosed due with alcohol abuse or dependence by the intake psychiatrist according to the Diagnostic and Statistical Manual of Mental Disorders-III (DSM-III) (Angold and Costello 1993) criteria as well as more advanced diagnosis tools. Also a mini mental status examination (Bertolucci et al. 1994) was conducted on all subjects and no memory deficit was observed. The alcoholic subjects had a history of heavily drinking for a minimum of 15 years. At least 30 days before the start of the experiment alcoholic subjects were hospitalized and fully detoxified.

Subjects were shown a series of object pictures chosen from the 1980 Snodgrass and Vanderwart picture set (Snodgrass and Vanderwart 1980). In this experiment, two picture stimuli appeared in succession with a 1.6 sec fixed interstimulus interval. The duration for the first (S1) and second (S2) picture stimulus in each test trial was 300 ms where the interval between each trial was fixed to 3.2 sec. All the pictures were paired either as matching or nonmatching conditions. For the matching condition, subjects were presented identical stimuli (similar S1 and S2) and in the nonmatched condition S2 was different from S1. Subjects were required to press a mouse key in one hand for the matching condition and press the mouse key in another hand for the nonmatching condition. The data were captured by an EEG device with 64 electrodes of which two were mounted for Electrooculography (EOG) and one nose electrode. We excluded these three channels as well as one more channel that had been used for grounding the subjects and only used data from 60 electrodes. More information regarding the data collection procedure can be found in reference (Zhang et al. 1995).

### Population-specific-data set

The first step of our methodological approach is to construct the Population-Specific-Data set (PSD). Population-specific-data set is composed of the elements that are computed by averaging the corresponding ERPs from the subjects within the population. The subspace spanned by the elements of the PSD provides generic brain functionality for the corresponding population over certain areas of the brain. We refer to the set of these subspaces generated by every element of the PSD as Population-Specific-Subspace (PSS).

In particular, suppose that there are *n* subjects that belong to *r* distinct populations. Also, let's assume that for every subject, there are *m* ERPs, each a *d*-dimensional time series. We denote the *i*th ERP of a subject by

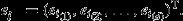


First, the *r* Population-Specific-Data sets (PSD) is computed from the ERPs corresponding to the subjects in every population. The PSD for population *j* shown as PSD_*j*_ contains *m* elements, where each element is a *d*-dimensional time series. The *i*th element of the PSD_*j*_, 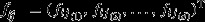 is obtained by averaging the *i*th ERPs from the subjects that belong to population *j* as below:

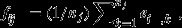
1where *n*_*j*_ is the number of subjects in population of *j* and *e*_*i,k*_ is the *i*th ERP corresponding to the *k*th subject of population *j*.

We consider the subspace (Halmos 1947) spanned by ***f***_***ij***_,

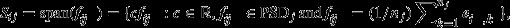
2as a set that represents a generic brain functionality for the corresponding area of the brain. We show the set of these subspaces generated by the elements of the PSD_*j*_ by PSS_*j*_. The subspace *S*_*ij*_ is paired with another subspace called orthogonal complement subspace (Halmos 1947).

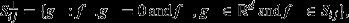
3which is composed of elements that are orthogonal and dissimilar to the elements of S_*ij*_. Thus, the subspace 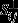 provides a generic representation for the brain functionalities with the maximum dissimilarity to the current elements of PSS_*j*._

### Orthogonal complement projection

The second step of our approach is to project the ERPs of a subject onto the orthogonal complement subspace of the PSS elements. The existing orthogonality between *S*_*ij*_ and 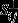 suggests that for the given population *j*, the affiliation of the ***e***_***i***_ to one of the other populations, say *j*′ ≠ *j*, would be more evident once ***e***_***i***_ is projected onto 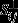. Generally, the projection for an arbitrary vector in 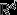 onto the orthogonal complement of the span(**E**)^⊥^ (**E** is an arbitrary matrix) can be obtained by constructing a projector 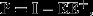
4where **E**^+^ = (**E**^**T**^**E**^−1^ − **E**^T^ represents the pseudo inverse of **E** (Greville 1960). Also, **EE**^+^ will be idempotent (i.e. ((**EE**^+^)^2^ = **EE**^+^)**,** and symmetric (i.e. (**EE**^+^)^T^ = **EE**^+^) (Heath 1997). By replacing

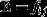


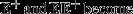


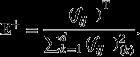
5

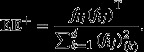
6

Hence, projector

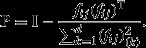
7would project a given vector onto the

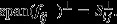


The projection of ***e***_***i***_ onto 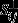 is given by

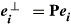


To quantify the similarity of ***e***_***i***_ to the population ***j′*** the cosine similarity between the 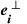 and ***f***_***i,j*****′**_ is computed as

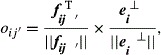
8where ||·|| correspond to norm-2 of the vectors (Peressini 1967).

An RBS vector with respect to population ***j′*** is composed of *m* o_*ij*′_ (*i* = 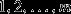) components. A component for specific *i* quantifies the similarity of an ERP of the subject to the corresponding *i*th element of the PSD_*j′*_. As it is explained in the Results section, we evaluate the efficacy of RBS vectors by using its components in a classification procedure as feature vector to classify alcoholic subjects from control subjects.

## Results

### Constructing relative brain signature vectors

As discussed in the method section, the data set contains 120 subjects of which 77 belong to alcoholic and 43 belong to the control populations. First, for every subject, an ERP data set is constructed by averaging the EEG signals corresponding to every channel across all different trials of the experiment. Then for every population, a PSD, namely Alcoholic-PSD and Control-PSD, is constructed. Every element of the PSDs, Alcoholic-PSD and Control-PSD, are computed by averaging the corresponding ERPs from the subjects in the alcoholic and control populations, respectively. The Alcoholic-PSD and Control-PSD is composed of 60 elements. We consider the subspace spanned by an element of PSD (see Methods section), which provides a generic brain functionality for a specific region of the brain. We refer to the subspaces generated by Alcoholic- and Control-PSDs as Alcoholic-PSSs and Control-PSSs, respectively.

Our proposed RBS feature extraction technique obtains information from subjects' ERPs by considering their relationship to both alcoholic and control populations. First, the subject's ERP is projected onto the orthogonal complement subspace (see Methods section) associated with the Alcoholic- and Control-PSS. The orthogonal complement subspace of the Alcoholic-PSS (Control-PSS) is a subspace in which elements illustrate distinctive functionality from the elements of the Alcoholic-PSS (Control-PSS). Hence, projection of subject's ERP onto the orthogonal complement subspace related to Alcoholic-PSS or Control-PSS contains transformed ERP components that signify subject's ERP association to the alcoholic or control populations. Each ERP is projected onto the orthogonal complement of the Alcoholic-PSS and onto the orthogonal complement subspace of the Control-PSS. To quantify the similarity of the projected ERP to the opposite population, the cosine similarity (see Methods section) between the projected ERP and the corresponding element from alcoholic and control population's PSS element is computed. These two resulting scalars explain the association of an ERP to the alcoholic and control populations. Therefore, by applying this procedure to all of the 60 ERPs two vectors of dimension 60 corresponding to the alcoholic and control population, namely Alcoholic-RBS and Control-RBS are generated. These vectors are an ordered collection of 60 elements which are called components. A component of the RBS vector depicts the similarity of an ERP associated with an area of the brain to the alcoholic or control population.

**Figure 1:**
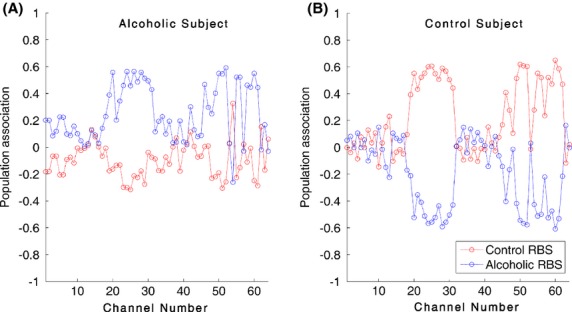
RBS vectors for an alcoholic and a control subject are illustrated. Two RBS vectors for an alcoholic subject and a control subject are shown. The components of the RBS vectors, quantify the association of an ERP waveform and the alcoholic population (shown in blue) or to the control population (shown in red). A positive value closer to 1, for a component of the RBS vectors indicates a stronger association to a certain population whereas smaller positive values and the negative values suggest the corresponding ERP data are weakly associated with a population. In A, majority of Alcoholic-RBS component values are significantly associated with the alcoholic population while for the Control-RBS component values, majority of the ERPs were weakly associated with the control population. In B, Control-RBS component values illustrate a strong relation to the control population while Alcoholic-RBS demonstrates weak association to the alcoholic population.

Relative-brain-signature vectors for an alcoholic and a control subject are illustrated in Figure1 by the dashed line that connects the computed RBS vector components for an alcoholic (Fig.1A) and a control subject (Fig.1B). The component values, which quantify the connection between an ERP and the alcoholic or control populations, are shown in blue and red, respectively. The RBS component value closer to one indicates a stronger association to a certain population whereas smaller positive values and negative values suggest that the corresponding ERP of the subject is weakly associated with a population. It is worth noting that the largest similarity values for the Alcoholic-RBS of an alcoholic subject are expected to be observed among the ERPs originating from the regions with the foremost distinctive functionality due to prolonged alcohol effects.

### Identifying Functionally Distinct Brain Regions of Alcoholic Subjects

In this section, the areas of the alcoholics' brain with distinct functional activity are identified using the RBS vectors. These areas are detected by exploring the corresponding brain regions associated with the RBS components. Considering that larger component values for the alcoholics' Alcoholic-RBS components are expected to be observed among the ERPs originated from the regions with distinct functional activity. We are particularly interested in identifying these components. A set of components for which large association value to the alcoholic population (i.e. large Alcoholic-RBS value) and small association value to the control population (i.e. small Control-RBS values) across alcoholic subjects is obtained. This set of component is associated with a set of electrode positioned on the areas of the brain with highest contribution in distinguishing between the alcoholic and control subjects. We attempt to determine these components through a classification procedure for classifying alcoholics from the control subjects. The classification procedure is composed of 60 classification experiments. For every classification experiment, feature vectors with a certain size are constructed from a set of RBS vector components. We compute the classification accuracy for every classification experiment and select the set of components for which the largest accuracy value is obtained. The details of the *i*th (*i* = 1, 2, … 60) classification experiment are explained in the following paragraph.

For a classification experiment, two-thirds of the alcoholic and two-thirds of the control subjects are randomly selected for training purposes and the rest of the subjects are selected for the testing purposes.

To construct the feature vectors, RBS components are weighted and sorted by using the RBS vector of the alcoholic subjects within the training set, using equation 1960, 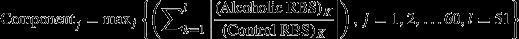
9where “|·|” is the absolute value, and *l* is the number of alcoholic subjects from the training set (i.e. since two-thirds of the alcoholic subjects are used for training the classifier, *l *=* *51). For every subject, the RBS vectors are resampled by keeping their first *i* ranked components and discarding the rest of the components' data (i.e. the sampled vectors contain *i* elements). Then, feature vectors for every subject are extracted by subtracting the resampled Control-RBS from the resampled Alcoholic-RBS. It is worth emphasizing that the components are ranked and selected according to the RBS vectors of the alcoholic subjects within the training set to avoid the double dipping phenomenon (Kriegeskorte et al. 2009).

To classify the subjects using the constructed feature vectors, Linear Discriminant Analysis (LDA) (Welling 2005) classification algorithm is employed. The generalization performance of every classification experiment is assessed by random subsampling in which the process of randomly partitioning subjects into training and testing sets is repeated many times (1000 in this study). For every classification experiment, we report accuracy, specificity, and sensitivity (Pang-Ning et al. 2006). The overall accuracy, specificity, and sensitivity values for every classification experiment are determined by averaging the accuracy, specificity, and sensitivity values computed for every run of the random subsampling procedure.

**Figure 2:**
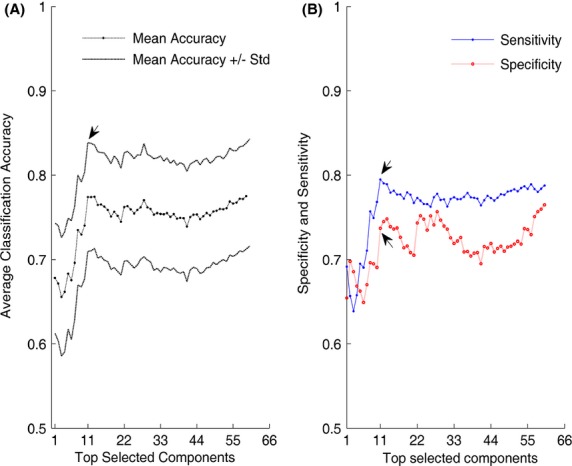
Performance evaluation for LDA classification between alcoholics and control subjects. (A) The *x*-axis corresponds to different number of significant components used to generate the feature vectors and the *y*-axis denotes the accuracy. The dot-line corresponds to the average classification accuracy and the dash-lines represent the standard deviation for the computed accuracy. (B) The *x*-axis corresponds to different number of significant components used to generate the feature vectors. The red and blue dot-lines denote the average specificity and average sensitivity (respectively) for a certain number of significant components used to construct the feature vector.

In Figure2, performance of the LDA classification algorithm is illustrated where different number of significant components is used to generate feature vectors. A dot in the dot-line of Figure2A corresponds to the average classification accuracy for a feature vector constructed from certain number of significant components. The dash lines in Figure2A represent the standard deviation for the computed accuracy. Dots in the red and blue dot-lines in Figure2B denote the average specificity and average sensitivity (respectively) for the corresponding significant components used to construct the feature vector. Starting with the first significant component, an accuracy of 0.67 ± 0.06, specificity of 0.65 and sensitivity of 0.69 are obtained. As it can be seen in Figure2, when the top 11 significant components of the RBS vectors are used to generate the feature vectors, the average accuracy, specificity, and sensitivity increase to their maximum values where the graphs' knee is formed. The highest classification accuracy value when the feature vector is constructed using the first 11 significant components is 0.78 ± 0.06 whereas the highest specificity and sensitivity for the first 11 significant components are 0.74 and 0.79, respectively.

**Figure 3:**
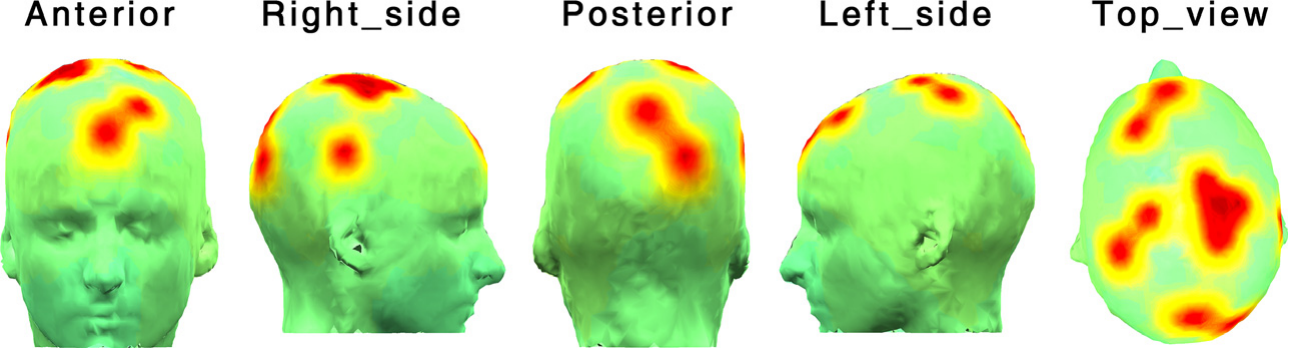
Different views for the top 11 functionally distinct brain areas between alcoholic and control subject. The red area corresponds to the most significant component and the yellow strip around the red determines the boundary of that region. These areas, with respect to their spatial extent are frontal and anterior frontal, centro-parietal, parieto-occiptal, and occipital lobes.

In Figure3, spatial distribution of the first 11 significant components over the scalp is illustrated using EEGLAB (Delorme and Makeig 2004). The red area corresponds to the most significant component and the yellow strip around the red determines the boundary of that region.

To investigate the consistency of the feature sets that are employed for the classification experiments across the sampling iterations, we report the sets of components with which feature vectors for the first 11 classification experiments are constructed. Due to a slight variability in the feature set across the 1000 iterations, the fraction of times that a feature set has been used for a classification experiment is denoted by a percentage value. As it is show in Table 2012, the feature sets selected for every classification experiment across the 1000 iterations are consistent (i.e. majority of the classifications have been performed with the similar set of features) for all the reported classification experiments.

**Table 1:** Sets of EEG channels (RBS components) selected for the first 11 classification experiments across their 1000 sampling iterations

Number of components used to construct the feature set	Selected components (EEG channel ID#)	Fraction of selection (in percentage)
1	[17]	100%
2	[17, 21]	94%
3	[17, 21, 45]	90%
4	[17, 21, 45, 48]	90%
5	[17, 21, 45, 48, 38]	90%
6	[17, 21, 45, 48, 38, 57]	89%
7	[17, 21, 45, 48, 38, 57, 5]	89%
8	[17, 21, 45, 48, 38, 57, 5, 52]	88%
9	[17, 21, 45, 48, 38, 57, 5, 52, 53]	89%
10	[17, 21, 45, 48, 38, 57, 5, 52, 53, 29]	88%
11	[17, 21, 45, 48, 38, 57, 5, 52, 53, 29, 12]	89%

EEG, Electroencephalography's

RBS, Relative-Brain-Signature.

## Discussion

Our proposed feature extraction approach of projecting the ERPs onto the orthogonal subspaces of the control and alcoholic populations has provided accurate details regarding the subjects' original population association. We extracted two 60-dimensional RBS vectors for every subject to characterize the relationship of the subject to the two populations of alcoholic and control. As Figure1A illustrates, the Alcoholic-RBS component values for an alcoholic subject signify a strong associated with the alcoholic population while majority of the Control-RBS component values of the ERPs, demonstrate a weak association to the control population. In Figure1B, the Control-RBS component values for a control subject illustrate a strong link to the control population while Alcoholic-RBS demonstrates a weak association to the alcoholic population. It suggests that the RBS vectors can potentially explain and distinguish the association of the subject to different populations of a study. We were also able to employ the RBS vectors to detect and visualize the functionally distinct regions over the alcoholics' brain. Among these areas, we identified a set of distinct regions from which the classification between alcoholic and control subjects gained its maximum accuracy (Fig.2A). In other words, these regions represent the functionally impaired regions of the alcoholics' brain and can be used as biomarkers to distinguish between alcoholic and control subjects. Figure3 provides scalp topography for these regions which correspond to the first 11 significant components. These areas, with respect to their spatial extent are frontal and anterior frontal, centro-parietal, parieto-occiptal, and occipital lobes. The distribution of these regions over the scalp indicates that the impact of the alcohol in the cerebral cortex of the alcoholics is spatially diffuse. The largest identified area (the centro-parietal area), engages more regions of the frontal lobe and right hemisphere relative to the left hemisphere which complies with the findings in other studies (Ellis and Oscar-Berman 1989; Zhang et al. 1997; Moselhy et al. 2001; Harris et al. 2008). The identified regions encompass a set of smaller areas that are known to be affected by ingesting one or two drinks of alcohol by social drinkers (Luchtmann et al. 2013). In other words, it seems that impairments caused by one or two (alcoholic) drink consumption by social drinkers targets the same regions of the brain that have are affected in the abstinent alcoholics.

**Figure 4:**
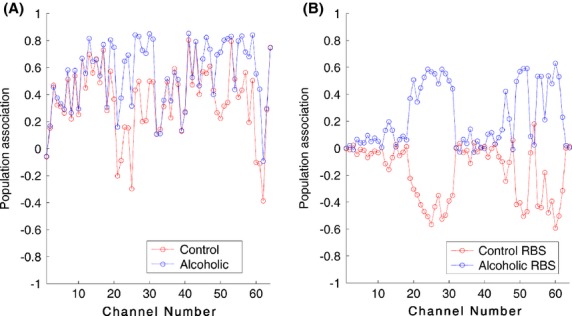
Process of computing RBS vectors without projecting signal onto the orthogonal subspaces and versus our proposed approach, for an alcoholic subject. (A) Subject's ERPs were not projected to the orthogonal subspaces and only similarity between subject's ERP and its corresponding ERP from Control-PSD and Alcoholic-PSD was computed. As demonstrated in a, very similar values of association to the alcoholic and control populations were obtained across all of the components of the Alcoholic- and Control-RBS. (B) The RBS vectors for the same subject were constructed by projecting the ERPs onto the orthogonal subspaces of the populations and then the similarity was computed. The computed similarity vectors in a were not able to characterize subjects with respect to its original population association in comparison to the RBS vectors illustrated in B.

We validated the efficacy of our novel technique, by repeating the process of computing the RBS vector, without projecting ERPs onto the orthogonal subspaces and only quantified similarity of the subject's ERP to the alcoholic and control subspaces. Figure4 shows the vectors composed of these values for an alcoholic subject. For computing similarity vectors, in Figure4A, ERPs are not projected onto the orthogonal subspaces and only the similarity between a given ERP and its corresponding ERP from Control- and Alcoholic-ERP is computed. As demonstrated in Figure4A, similar values of association to the populations are obtained across all of the components of the alcoholic- and control-similarity vectors. In Figure4B, the RBS vectors for the same subject are constructed by projecting the ERPs onto the orthogonal subspaces of the populations and then the similarity is computed. The computed similarity vectors (Fig.4A) are not able to characterize subjects with respect to its original population association in comparison to the RBS vectors illustrated in (Fig.4B). This result verifies our underlying assumption that the orthogonal complement subspaces of a PSS element provides a generic domain to represent distinctive brain functionality for populations other than the population of PSS. It should be emphasized that in this work, when evaluating population association of a subject, we investigated the similarities and dissimilarities for the entire period of the experiment and disregarded exploring the similarities (or dissimilarities) in the smaller temporal windows. However, our proposed technique is not bounded to analysis of the ERPs for a long period of time and can be used to reveal the potential dynamic pattern of an alcoholic's distinctive areas.

Finally, it is worth mentioning that our approach enables distinguishing between any numbers of populations and is not limited only to two populations. It may also be used for intra-population classification given enough meta-information regarding subjects within a population. We will attempt to address this issue in our future studies by collecting more information regarding alcoholic subjects' mental and physical health to perform an intra-alcoholics classification experiment.
